# PVJ1 Is Not the First Tailed Temperate Phage Infecting Bacteria from Genus *Psychrobacillus*. Comment on Liu et al. Isolation and Characterization of the First Temperate Virus Infecting *Psychrobacillus* from Marine Sediments. *Viruses* 2022, *14*, 108

**DOI:** 10.3390/v14030495

**Published:** 2022-02-28

**Authors:** Nikita Zrelovs, Kristaps Lamsters, Janis Karuss, Maris Krievans, Andris Dislers, Andris Kazaks

**Affiliations:** 1 Latvian Biomedical Research and Study Centre, Ratsupites 1 k-1, LV-1067 Riga, Latvia; nikita.zrelovs@biomed.lu.lv; 2 Polar Research Center, Faculty of Geography and Earth Sciences, University of Latvia, Jelgavas 1, LV-1004 Riga, Latvia; kristaps.lamsters@lu.lv (K.L.); janis.karuss@lu.lv (J.K.); maris.krievans@lu.lv (M.K.)

In a recent study published in *Viruses* [1], the authors claim to have isolated and characterized the first tailed temperate phage able to infect a bacterium from the genus *Psychrobacillus*—*Psychrobacillus* phage PVJ1 (GenBank accession number: MZ983385). These claims, however, are misleading, as at least two tailed temperate siphophages infecting a *Psychrobacillus* sp. had their complete annotated genomes deposited in public biological sequence repositories prior to the public availability of phage PVJ1.

Based on the respective GenBank submission associated with the aforementioned paper, the complete annotated genome of *Psychrobacillus* phage PVJ1 (MZ983385.1) was deposited to GenBank on 31 August 2021 and subsequently released on 22 September 2021, while the complete annotated genomes of *Psychrobacillus* phages Perkons (MT325768.1) and Spoks (MT410774.1) were deposited to GenBank on 10 April 2020 and 29 April 2020, and were publicly released on 6 May 2020 and 27 May 2020, respectively, more than a year prior to phage PVJ1.

Although the authors performed a great job at describing the isolation and characterization of *Psychrobacillus* phage PVJ1, we found it quite disturbing that they, apparently, have not attempted a quick public biological sequence repository search using a query as simple as “*Psychrobacillus* phage” prior to claiming to have reported the first known phage infecting a member of the genus *Psychrobacillus*. This has prompted us to highlight the necessity of having a look into the International Nucleotide Sequence Database Collaboration (INSDC) databases (e.g., GenBank, European Nucleotide Archive, DNA Data Bank of Japan) before making any claims regarding newly isolated and characterized phages’ place within the context of the known phage diversity (such as claiming that the phage in question is the first known virus to infect the members of bacterial genus “X”).

To our knowledge, phages Perkons and Spoks are the first phages capable of infecting a bacterium belonging to the genus *Psychrobacillus* that had their complete annotated genomes publicly available. These phages, along with their host—*Psychrobacillus* sp. L4—were retrieved in the Latvian Biomedical Research and Study Centre from ice-free Antarctic soil material collected during the First Latvian Antarctic Expedition (K. Lamsters, J. Karuss, M. Krievans) that took place from 18 February to 4 April 2018 and received by us in the summer of the same year. Even though not formally published in the peer-reviewed literature yet, the process of isolation and characterization of these phages has been a major part of N. Zrelovs’ MSc. thesis on the isolation and characterization of phages for bacterial genera, which had no known phages able to infect them yet, so as to expand the known cultured phage diversity, defended at the University of Latvia in summer 2020 [2]. The preliminary findings of these studies (including the presentation of the employed methodology, EM micrographs and annotation of a draft genome assembly for “*Psychrobacillus* virus L4F1” later to be refined and deposited to GenBank as “*Psychrobacillus* phage Perkons” under accession number MT325768.1) were also presented in the form of an oral presentation, “Isolation and characterization of novel bacteriophages from Antarctic soil samples”, at the “Bioresources and Viruses IX” conference held in Kyiv, Ukraine (9–11 September 2019; presentation enclosed within the letter as Supplementary S1).

The authors, apparently, have isolated and characterized the first known temperate myovirus capable of infecting a *Psychrobacillus* sp.—phage PVJ1—whose native host *Psychrobacillus* sp. GC2J1 came from marine sediments. However, overall, the phage PVJ1 still remains the third temperate tailed virus infecting a member of this bacterial genus following the temperate *Psychrobacillus* siphophages Perkons and Spoks, despite the fact the authors claim that “… there were no reports of bacteriophages infecting the genus *Psychrobacillus* …”, “… PVJ1 is the first bacteriophage isolated from *Psychrobacillus*” and “… PVJ1, the first-tailed temperate phage-infecting *Psychrobacillus*” within the paper [1]. Interestingly, although the host of phages Perkons and Spoks was initially classified by us based on 16S rRNA gene near-full-length sequencing as a strain of *Psychrobacillus psychrodurans* (on the basis of the closest 16S rRNA gene sequence match from cultured bacteria), the genus seems to have expanded shortly after with the addition of species that are more closely related to *Psychrobacillus* isolate L4—*Psychrobacillus vulpis* [3] and *Psychrobacillus glaciei* [4]—with the latter being isolated from an Antarctic iceberg and having the closest 16S rRNA gene sequence match to isolate L4 as of now (Figure 1).

The growing accessibility of next-generation sequencing has remarkably impacted the rate of novel phage isolation and characterization studies conducted worldwide, resulting in a rapid expansion of the known phage diversity via steady growth of the complete phage genomes available in INSDC databases, albeit mostly with phages infecting a very limited number of different hosts. Although the peer-reviewed literature contains some attempts at summarizing the complete phage genome contents of the public databases (i.e., our attempt at doing so [11]), such studies, logically, provide no more than overview snapshots of the uncovered phage diversity at a given time and are always prone to becoming obsolete in the light of such diversity being ever-growing. To allow following the expansion of GenBank with novel phage entries, recently, a methodological framework that allows taking such “phage diversity snapshots” and even additionally extracting meaningful data for further phage comparative genomics analyses (INPHARED) was developed and published by Cook and colleagues [12] but requires some command line proficiency from the potential user seeking to run it independently of the (thus far monthly) output updates by the authors, making manual public database searches still a necessity for some researchers.

Despite the fact that there is a large fraction of complete annotated phage genome entries publicly available that still do not have an article or at least a genome announcement from the peer-reviewed literature linked to them for a variety of reasons (including *Psychrobacillus* phages Perkons and Spoks), we believe it is incorrect to ignore the existence of such entries when analyzing the place of any newly isolated phage within the context of known phages, regardless of the phages not being mentioned anywhere in the peer-reviewed literature. With this letter, we would like to emphasize the self-evident need of phage researchers to make use of public biological sequence repositories to keep track of the currently available phage diversity, especially for supplementing claims requiring a high burden of proof (e.g., the isolated phage is the first known phage infecting “X”, where “X” is a variable). As is exemplified by the case of the study describing the isolation and characterization of *Psychrobacillus* phage PVJ1, failure to do so might result in incorrect and misleading conclusions regarding the place of newly isolated phages within the thus far uncovered phage diversity, which is inevitably going to expand further beyond the currently recognized state, which still encompasses only a very limited variety of distinct hosts as of today.

## Figures and Tables

**Figure 1:**
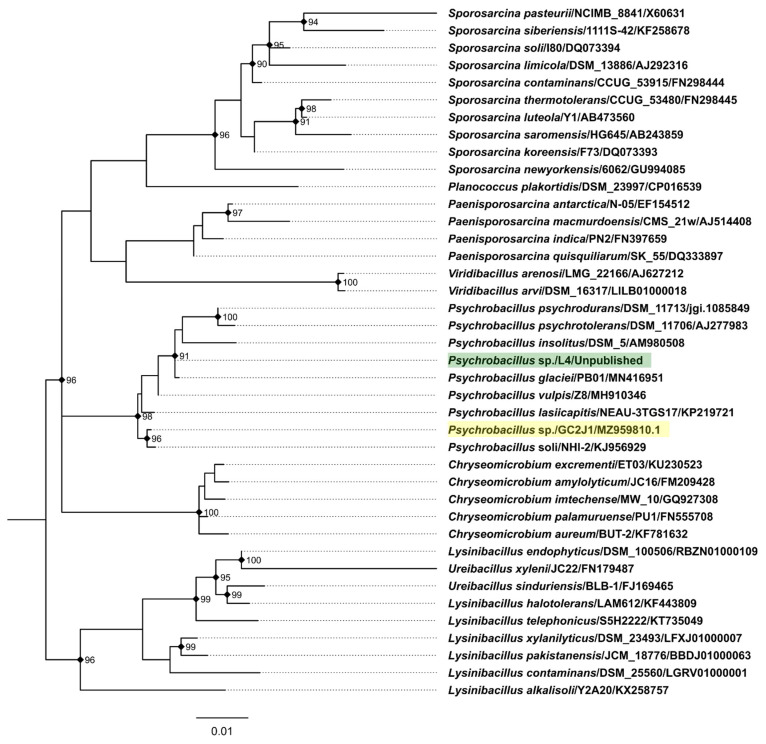
Maximum likelihood phylogeny of the partial 16S rRNA gene sequences of *Psychrobacillus* isolate L4 (host of phages Perkons and Spoks; tip label highlighted in green; sequence obtained using common 27F and 1492R primers), *Psychrobacillus* isolate GC2J1 (host of phage PVJ1; tip label highlighted in yellow) and validly named closely related bacterial isolates found in the EzBioCloud database [5]. Clustal-Omega v.1.2.4 [6] was used in auto mode for the multiple sequence alignment (MSA), which was then restricted to informative positions only using Gblocks [7] under default settings, resulting in the MSA of 40 sequences and 1250 positions (148 parsimony-informative, 87 singleton sites, 1015 constant sites). Maximum likelihood phylogeny reconstruction was performed using IQTREE v2.0.6. [8] under K2P+R2 as the best-fit substitution model according to ModelFinder [9] while allowing for polytomies. Ultrafast bootstrapping (UFBoot [10]; 1000 replicates) was used to assess the node support. The tree is midpoint rooted and drawn to scale; branch lengths are in the unit of nucleotide substitutions per site. UFBoot support percentages are given for nodes having a support of more than 90%; these are also indicated by black rectangles. Tip labels are in the form of “Species/Strain/Accession”.

## Data Availability

Complete annotated genome sequences of *Psychrobacillus* phages Perkons and Spoks are available at GenBank under accession numbers MT325768.1 and MT410774.1, respectively.

## Supplementary Materials

The following supporting information can be downloaded at: https://www.mdpi.com/article/10.3390/v14030495/s1, Supplementary S1: Slides accompanying the oral presentation “Isolation and characterization of novel bacteriophages from Antarctic soil samples” given by N.Z. at “Bioresources and Viruses IX” conference held in Kyiv, Ukraine (9–11 September 2019).
