# Supplementary material for: PVJ1 Is Not the First Tailed Temperate Phage Infecting Bacteria from Genus Psychrobacillus. Comment on Liu et al. Isolation and Characterization of the First Temperate Virus Infecting Psychrobacillus from Marine Sediments. Viruses 2022, 14, 108
Source: Viruses. 2022 Feb 28;14(3):495. doi: 10.3390/v14030495 (PMC8951557; doi:10.3390/v14030495)
Supplement: Supplementary file 1 [file viruses-14-00495-s001.zip › Appendix_S1_21-02-22.pdf]

Abstract

Isolation and characterization of novel bacteriophages from Antarctic soil samples

Ņ. Zrelovs1, E. Černooka1, K. Lamsters2, J. Karušs2, M. Krievāns2, A. Dišlers1, A. Kazāks1

1. Latvian Biomedical Research and Study Centre, Riga, Latvia.

2. University of Latvia, Faculty of Geography and Earth Sciences, Riga, Latvia.

(nikita.zrelovs@biomed.lu.lv)

Decades after its discovery in 1820, Antarctica, arguably the most desolated and roughest place on planet Earth, was thought to be a relatively sterile environment due to its scarce lifeform variety, however, continuous multidisciplinary research up to this date has unveiled that the continent is far from sterile [1]. First microbes in Antarctica were found circa 1900, but not until several decades later attempts to not only isolate, but also culture and identify them have been successfully made [2]. It is known that most existing bacteria cannot be cultured using traditional microbiological techniques, the observation that was termed "the great plate count anomaly"[3], but recent advancements in culture-independent microbial research approaches, emergence of metagenomics, in particular, have allowed to partly overcome this existing limitation and revealed even greater microbial diversity in various environmental samples, including Antarctic soils[4, 5]. Presence of microbial communities undoubtedly indicates the presence of corresponding bacteriohages as well, while genomes of bacteriophages have proven themselves to be a treasury of novel genes. Needless to say that many of these genes encode products for which various practical applications, most notably in the field of molecular biology, have been found [6]. Whereas metaviromics approach might be the fastest way to mine such genes from the environmental samples, it substantially limits study opportunities of individual phage microbiological characteristics which are of utter importance to expand the global bacteriophage diversity knowledge. Therefore, we would like to report our progress on the process of culture-based isolation, whole genome sequencing and further genomic characterization of novel bacteriophages, including a first known Sporosarcina sp. infecting phage, from samples collected from ice-free soils of Antarctic Peninsula.

Latvian Biomedical Research and Study Centre
research and education in biomedicine from genes to human

UNIVERSITY OF LATVIA
ANNO 1919

Isolation and characterization of novel bacteriophages from Antarctic soil samples

N. Zrelovs[1], E. Černooka[1], K. Lamsters[2], J. Karušs[2], M. Krievāns[2], A. Dišlers[1], A. Kazāks[1]

1. Latvian Biomedical Research and Study Centre, Riga, Latvia.

2. University of Latvia, Faculty of Geography and Earth Sciences, Riga, Latvia.

(nikita.zrelovs@biomed.lu.lv)

"Bioresources and Viruses IX"
September 9-11, 2019.
Kyiv, Ukraine

First Latvian Antarctic expedition

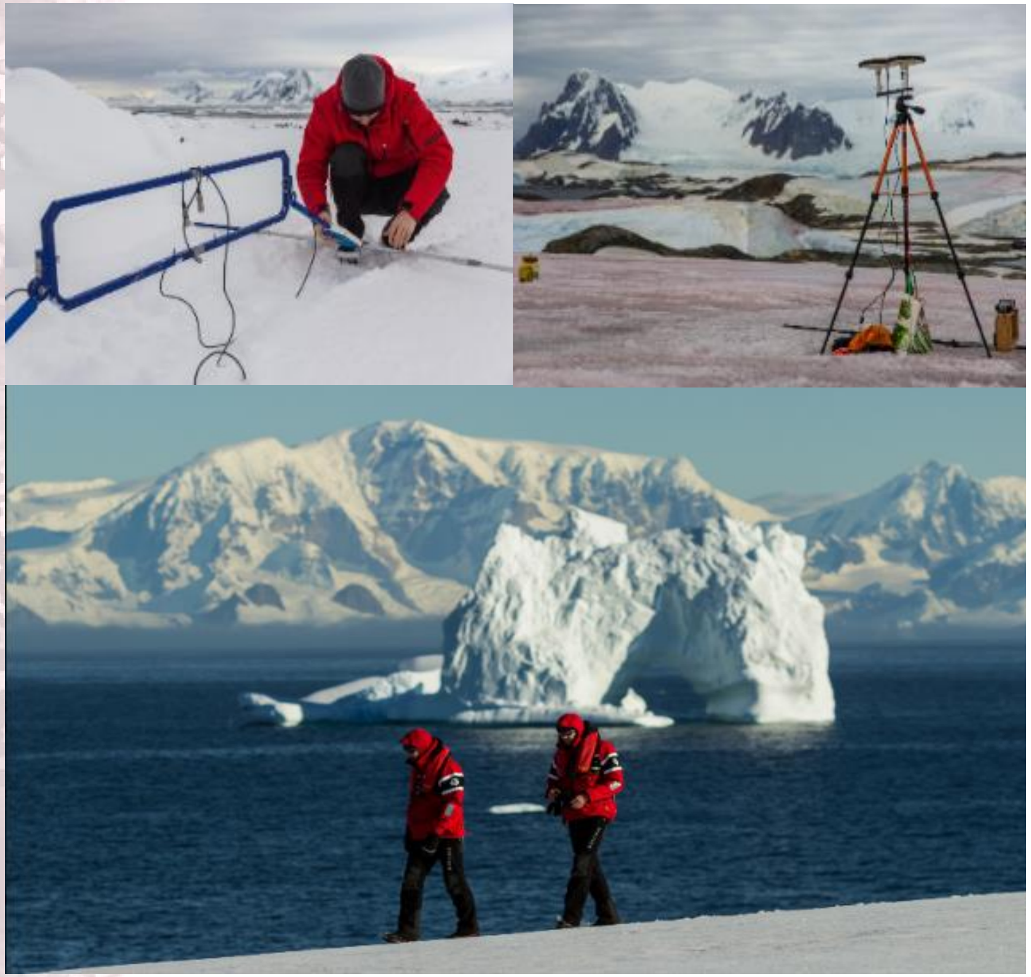

- The first Latvian Antarctic expedition (Scientists: K. Lamsters, J. Karušs, M. Krievāns), took place from February, 18 to April, 04, 2018.

Research goals:

- Studies of the inner structure and thickness of icecaps on Argentine Island
- Creation of orthophoto maps and digital elevation models of the Argentine Islands
- Antarctic soil sampling

UNIVERSITY OF LATVIA
ANNO 1919

Study area

The purpose of the sampling was to collect Antarctic soil samples to characterize soils and their development in the coastal part of the Antarctic Peninsula and surrounding Argentine Islands.

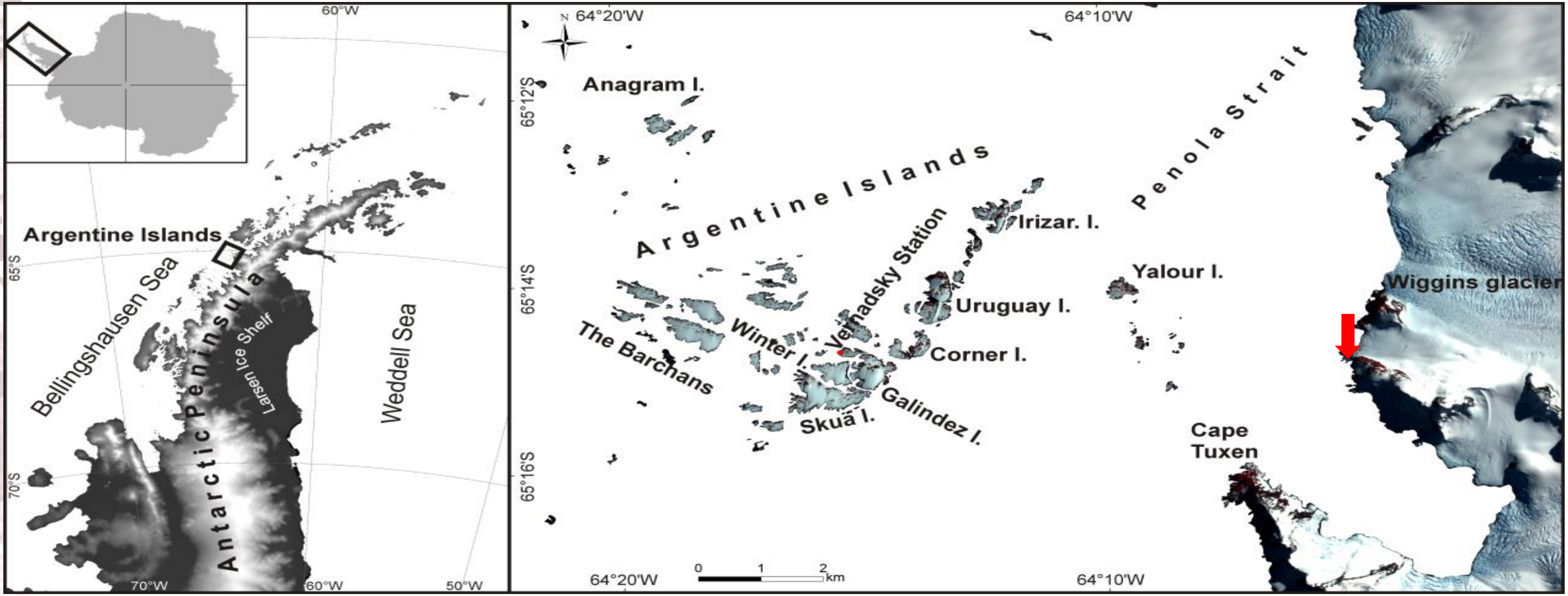

Soil sampling

- The soil was sampled on several islands and not far from the Rasmussen hut, Waddington Bay, Graham Coast, Antarctica.
- Three soil pits were excavated by shovel in one sampling place a few meters away from each other (small depression filled by swampy soil consisting mainly from remains of mosses).
- Samples were carefully taken form each soil horizon
  (0-4 cm; 4-10cm; 10-11.5cm)
  using a spoon and collected in plastic bags.
- Samples were stored at room temperatures for a few weeks.

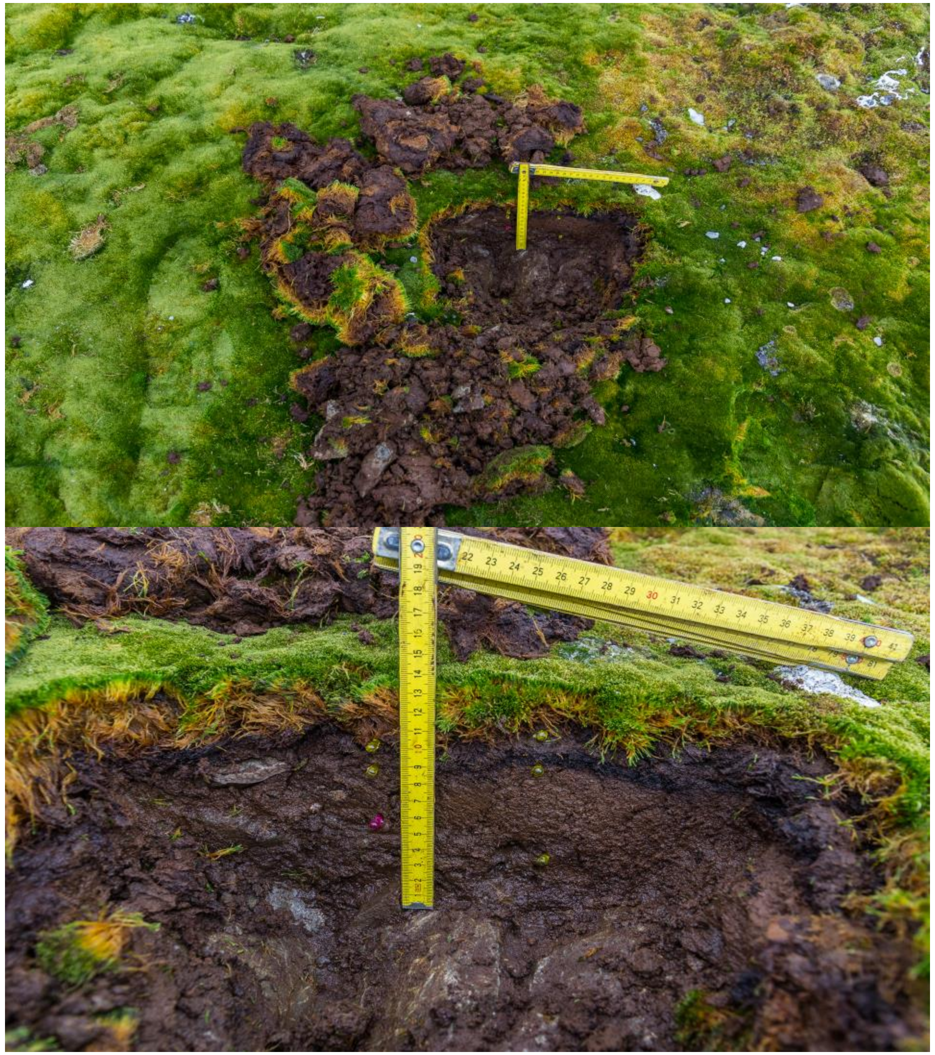

Host isolation

- 3 grams of soil in 50mL LB broth
- Shaken for 8h at 160rpm (RT)
- Culturing at +4°C
- Colonies of different morphologies isolated and further individually propagated on CR agar plates
- Genomic DNA extraction (MagaZorb® DNA Mini-Prep)
- 16s rRNA gene PCR (27F and 1492R universal primers)
- ~1450bp long PCR product excised from gel after NAGE (GeneJET Gel Extraction kit)
- Sanger sequencing (BigDye™ Terminator v3.1 Cycle Sequencing Kit)
- Bacteria taxonomic identification (16s rRNA BLAST, Ezbiocloud and rdbproject databases)

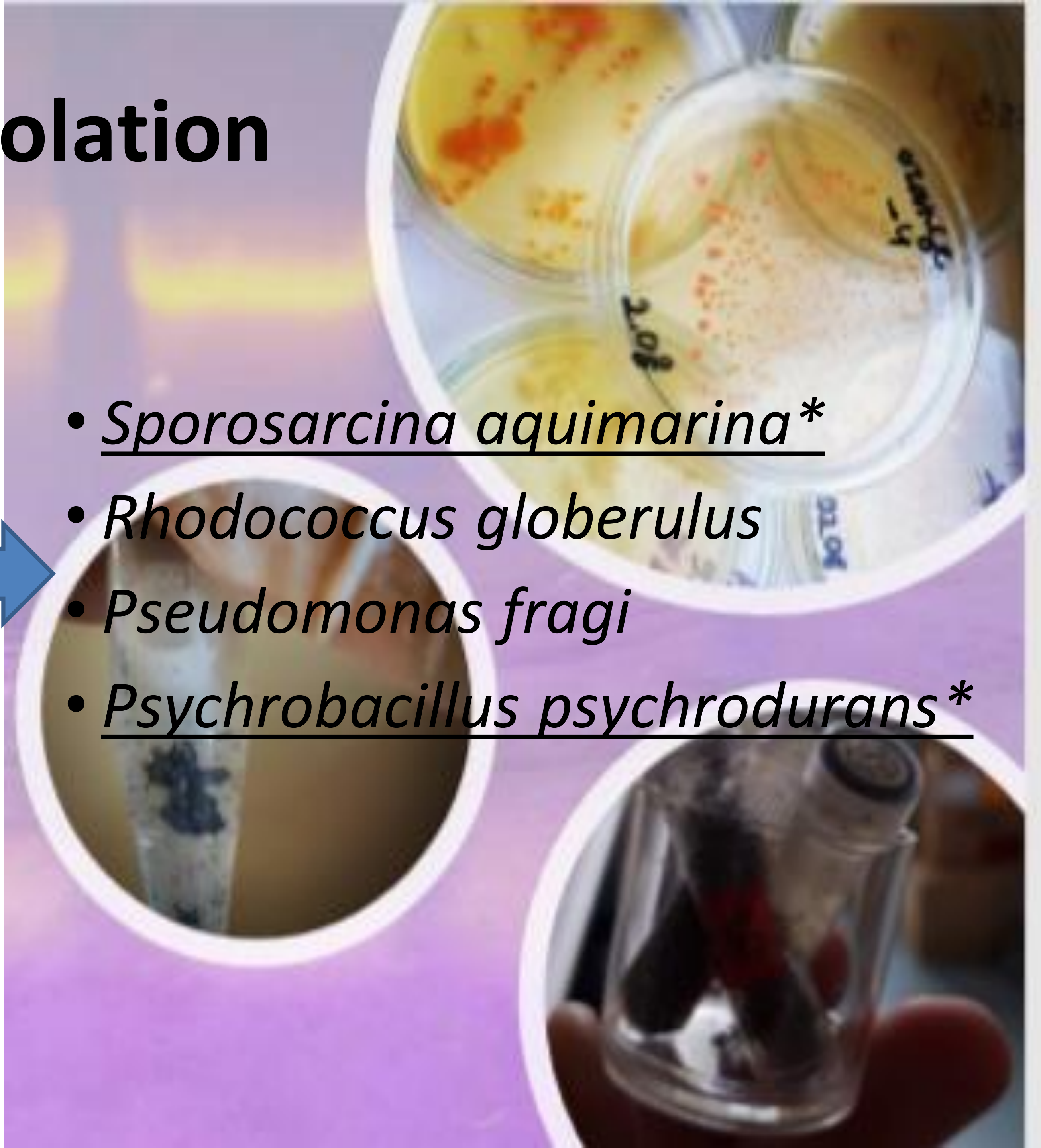

- _Sporosarcina aquimarina_*
- _Rhodococcus globerulus_
- _Pseudomonas fragi_
- _Psychrobacillus psychrodurans_*

Bacteriophage hunt

PRIMARY ISOLATION:

- 3 grams of soil in 100 mL LB broth (supplemented with 10 mmol l^{-1} $CaCl_2$ and 10 mmol l^{-1} $MgCl_2$)
- Shaken overnight at 150rpm (RT)
- Bacterial debris sedimented 10000 rpm for 30 min
- Supernatant filtered through 0.45 µm filter
- Spot tests on identified bacteria
- Double-agar overlay assays
- Plaque purifications
- Phage extracted from well-lysed bacterial lawns in soft agar (multiple plates)

PHAGE SCALE-UP PROPAGATION

- 200 mL of LB broth inoculated with freshly collected host cells
- Incubated on a shaker (150 rpm, RT) until OD_{540} has reached 0.3-0.4
- Infected with phage at MOI ~1
- Debris sedimented and supernatant filtered
- Phage particles were sedimented at 24000 rpm for 1 h using JA-30.50 Ti rotor on Beckman Coulter Avanti J-E centrifuge ~ 65000 RCF
- Jelly-like sediment was resuspended in ~4 mL of supernatant
- HiTrap Capto DEAE column chromatography of phage particles

DNA EXTRACTION AND SEQUENCING

- Treatment with Proteinase K and SDS (1h at +56°C)
- Random physical shearing with average fragment size of 550bp (Covaris S220 ultrasonicator)
- TruSeq DNA nano library prep and QC (Agilent 2100 Bioanalyzer, Qubit)
- WGS using Illumina's MiSeq platform (PE, 2x250)

Post-WGS

Raw data assessment and manipulation:

- FASTQC
- Trimmomatic
- bbnorm

de novo assemblies:

- SPAdes
- MIRA4

Assembly assessment:

- BWA/Bowtie2
- IGV viewer
- GeneStudio
- PhageTerm

REFINEMENT?

Annotation:

- Glimmer/GeneMark/Prodigal
- tRNA scan/Aragorn
- BLASTn/BLASTp
- HHPRED
- DNAmaster

“There's more than one way to do it”

Phage overview

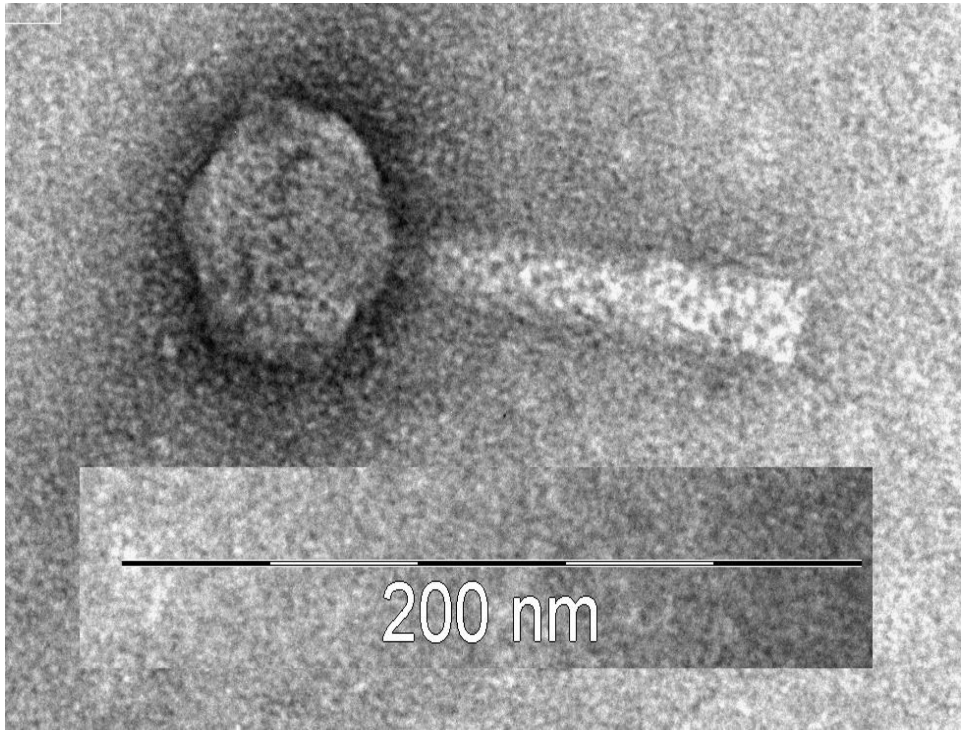

Figure 1. *Sporosarcina* virus A2F.

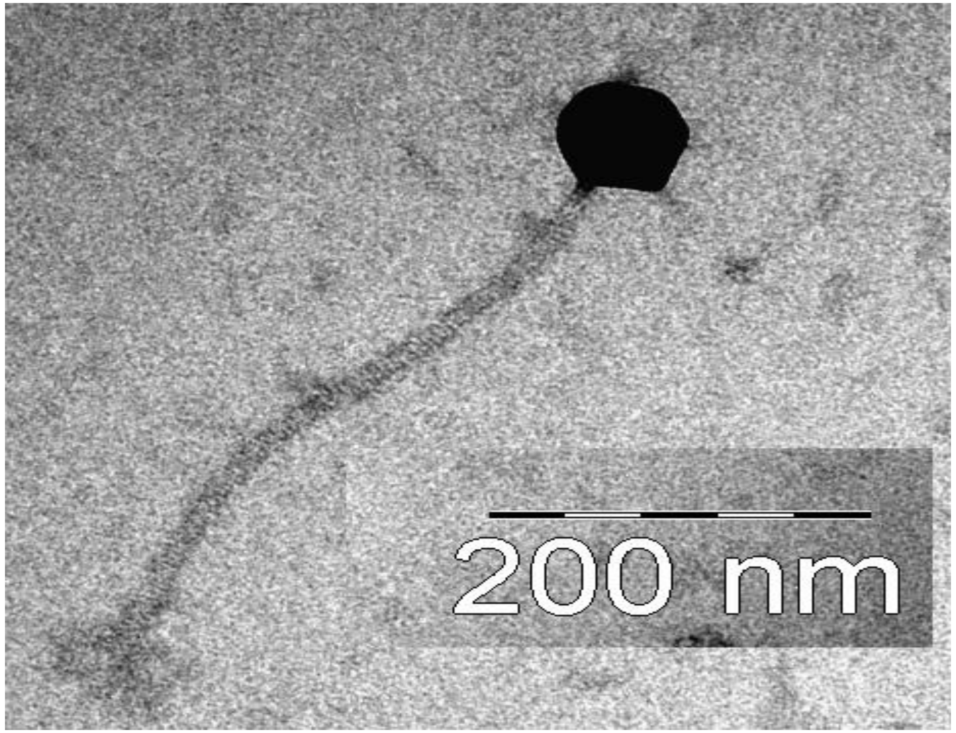

Figure 2. *Psychrobacillus* virus L4F1

Isolate name	Genome size (bp)	GC content (%)	Number of ORFs	Coding capacity (% of Genome Length)	Uniqueness*	Host (Gram-stain)	Morphology	Termini
L4F1	136938	30.60%	219	87.97%	99.24%	*Psychrobacillus psychrodurans* (+)	*Siphoviridae*	SDTR (264 bp)
A2F	64517	49.90%	101	*91.18%*	99.30%	*Sporosarcina aquamarina* (+)	*Myoviridae*	SDTR (437 bp)

* Nucleotide level uniqueness, as compared to "best" BLASTn hit (megablast option) . Uniqueness = 1 - (Query cover * Identity)

Genomic map of *Sporosarcina* virus A2F

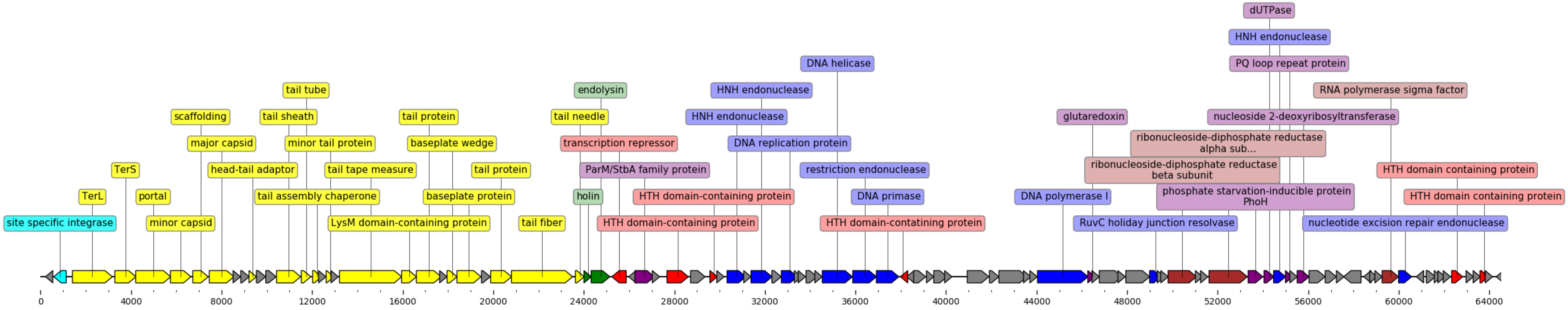

Yellow – Morphogenesis;
Blue – Replication and Repair;
Red – Transcription;
Green – Lysis;
Cyan – Lysogeny;
Grey – Unknown functions;
Purple – Additional functions;
Brown – RNA manipulation

- Functions for products of 52 (51.5%) out of total 101 ORFs remain without any putative functional prediction
- 91 ORF on forward (+) and 10 ORFs on reverse (-) strands
- Start codon usage: ATG – 76; TTG – 11; GTG – 14; CTG – 0

Genomic map of *Psychrobacillus* virus L4F1

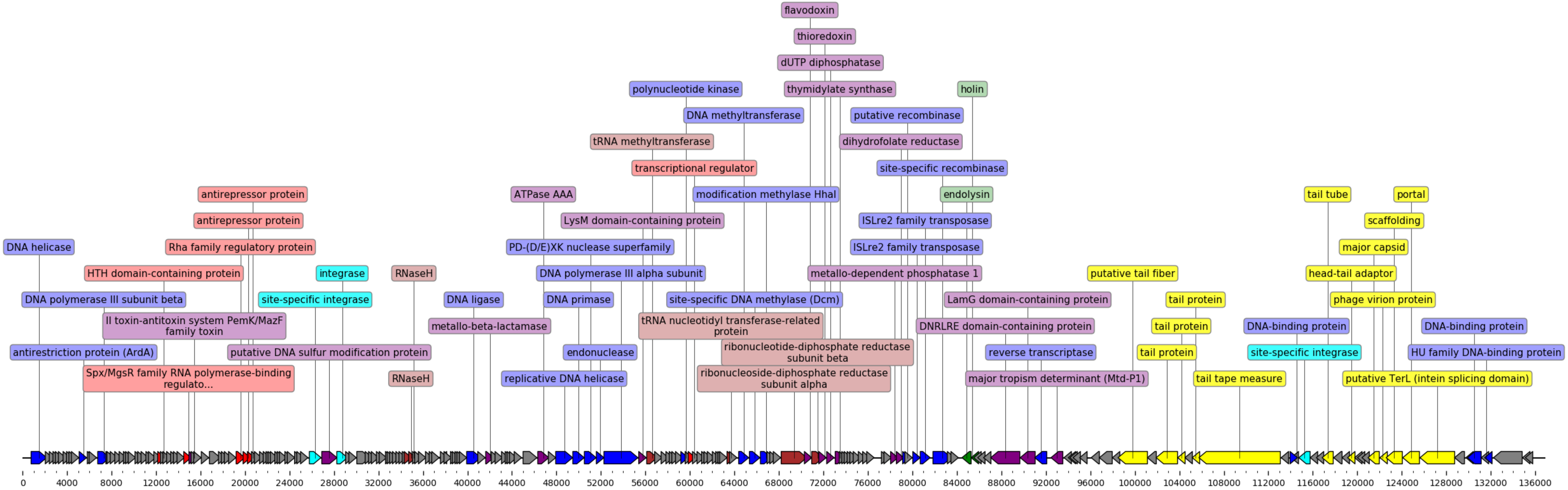

Yellow – Morphogenesis;

Blue – Replication and Repair;

Red – Transcription;

Green – Lysis;

Cyan – Lysogeny;

Grey – Unknown functions;

Purple – Additional functions;

Brown – RNA manipulation

- Functions for products of 154 (72.6%) out of total 212 ORFs remain without any putative functional prediction
- 153 ORF on forward (+) and 59 ORFs on reverse (-) strands
- Start codon usage: ATG - 177 TTG - 27 CTG - 0 GTG - 15

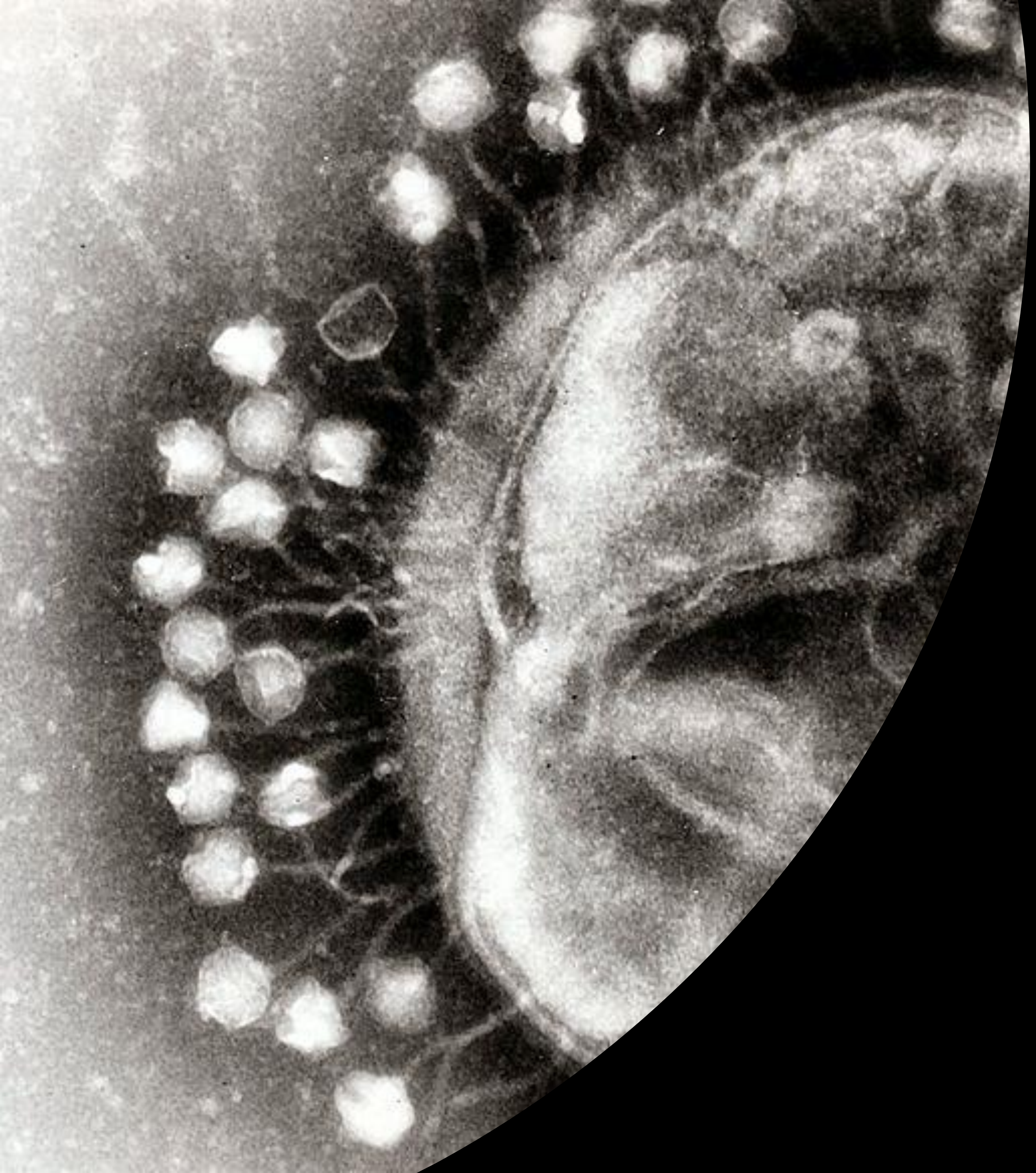

Thank you for your attention!
